# A public health call for action to address liver cancer and hepatitis in northern Ghana

**DOI:** 10.1038/s43856-025-00854-2

**Published:** 2025-04-16

**Authors:** Chloe Tuck, Abdul Rashid Timtoni Iddrisu, Salimatu Asam Moro, Robert Akparibo, Richmond Aryeetey, Laura Gray, Richard Cooper

**Affiliations:** 1 Sheffield Centre for Health and Related Research, Division of Population Health, School of Medicine and Population Health, University of Sheffield, Sheffield, UK; 2 School of Public Health, University of Ghana, Accra, Ghana; 3 Oncology, Tamale Teaching Hospital, Tamale, Ghana; 4 Gastroenterology, Tamale Teaching Hospital, Tamale, Ghana; 5 Fred Binka School of Public Health, University of Allied Health Sciences, Ho, Ghana

**Keywords:** Cancer prevention, Hepatitis B, Hepatitis C, Hepatocellular carcinoma

## Abstract

Tuck et al. discuss the unethical lack of access to diagnosis and treatment of hepatitis and consequential avoidable liver cancer cases. Existing evidence and current research in Ghana identifies a number of ways to address hepatitis and liver cancer in Ghana.

## Teaser

Lack of access to diagnosis and treatment of hepatitis is unethical, resulting in avoidable liver cancer cases. Drawing on existing evidence and current research in Ghana, we identify a number of ways to address hepatitis and liver cancer in Ghana.

## Introduction

April 2024 saw the World Health Organization (WHO) release of the *Global hepatitis report 2024: action for access in low- and middle-income countries*^1^. The growing burden of hepatitis globally is particularly concerning given it leads to a severely high likelihood of developing hepatocellular carcinoma (HCC)^2^. The World Hepatitis Alliance has called for hepatitis to be recognised for its role in causing cancer^3^ as many liver cancer cases could easily be prevented through screening and vaccination^4,5^. This is not dissimilar to cervical cancer, which the WHO has launched a plan to eradicate^6^. Hepatitis disproportionately causes liver cancer in Africa; in 80% (28/35) of African countries surveyed, over 70% of liver cancer cases are attributed to the infection^7^. However, a lack of epidemiological data in Africa means levels are likely underestimated^8^ as so many patients are being overlooked and underserved. We argue that countries such as Ghana bear a significant (but avoidable) burden and that several initiatives could be introduced relating to hepatitis and liver cancer.

## Hepatitis in Ghana

Although there are five main strains of hepatitis, hepatitis B and C represent the largest global disease burden^2^. In Ghana, it was estimated that there was 2,865,177 and 442,797 hepatitis B and C cases respectively in 2023^1^. This is of major concern as global estimates suggest 4 out of 5 cases are undiagnosed^4^. Without a registry and routine screening procedures at the population level, the true number of cases is hard to accurately assess in Ghana. Hepatitis is also associated with poverty as poor and marginalised groups have higher incidence globally along with reduced access to related health services^9^. The disease is often transferred during childbirth or in early childhood. Antenatal transfer of hepatitis B, increases the likelihood of chronic infection and leads to severe disease with early onset of liver cirrhosis and hepatocellular carcinoma (HCC)^10–12^.

## Liver cancer in Ghana

For both sexes, liver cancer was the second most common cancer in Ghana in 2020^13^. Moreover, it was estimated to cause 3,166 deaths per year in 2020 according to GLOBOCAN estimates, making it the largest contributor to cancer death in Ghana^13,14^.

District facilities reports from Northern Ghana suggest HCC caused by hepatitis is a growing problem and the largest cancer burden they face. In terms of case load, it may supersede breast cancer and cervical cancer, which have been raised to the public health agenda in recent years.

Hepatitis is typically diagnosed through serological tests for hepatitis surface antigens and rapid diagnostic tests are now being developed that reduce the sample needed and allow same day turnaround^15^. This has traditionally been managed through conventional antiviral therapies. However new therapeutic advances are being made through specific molecular targets that inhibit key proteins in the viral life cycle and silence replication using RNAi^15^. Early treatment is crucial to prevent development into liver cirrhosis and HCC^15^.

In Ghana most cases of HCC present at a late stage, only being eligible for supportive palliative care^16,17^. These outcomes are a result of several factors including poor awareness, delayed treatment seeking behaviours^16^ and patient preference for traditional herbal and spiritual treatments^18,19^. Many community level facilities lack training in HCC diagnoses, which means some cases are misdiagnosed with gastritis. In the case when patients may be eligible for other therapies, such as systemic therapy and resection, they struggle to afford the associated diagnostic tests, travel and treatment, leading to high levels of drop out against medical advice. Systemic therapies are not available on the national health insurance scheme and anecdotal evidence suggest this is highly expensive compared to the median monthly income (estimated at 2000 GHS/month, approximately £140 in August 2023)^20^. Liver resection is only available at major facilities in Accra and Kumasi.

## Initiatives to eradicate hepatitis C and prevent liver cancer

Attempts to curb HCC through tackling hepatitis C elsewhere on the African continent have seen some success. For example, Egypt has estimated to have diagnosed 87% of hepatitis C cases that exist and provided treatment to 93% of these through local drug manufacture^1,4^ indicating substantial gains in the disease’s eradication nationally. Following their success, the Egyptian government has partnered with Ghana to supply hepatitis C medicine^1^. Barriers to patients still remain, such as concerns over medication availability and pre-requisite testing prior to treatment eligibility. Lower-income groups and those in rural areas are disadvantaged as testing for hepatitis C in Ghana occurs on a walk-in basis and carries fees. Thus, this current treatment availability profile is only expected to increase the inequalities in hepatitis C status awareness and prevention.

## Initiatives to eradicate hepatitis B and prevent liver cancer

Hepatitis B cannot be cured but it can be prevented with vaccination and when detected, effectively managed to prevent development into HCC and antenatal transfer. Screening is not free in most African countries including Ghana. In addition to initial testing, diagnostic tests are required to ascertain stage and whether treatment is required. Treatment imparts high monthly cost on patients leading to high levels of drop-out. While screening of pregnant women is available, the vaccine is not routinely offered at birth as part of Ghana’s Expanded Programme on Immunisation (EPI), despite this been shown as an effective way to prevent transmission. When available, costs, and poor maternal and health professional understanding, may hinder uptake^21^.

## Conclusion

There is an imminent need to address the burden of hepatitis as a crucial strategy to combat liver cancer in Africa. Taking learnings from Ghana, strong preventative measures to detect, treat and vaccinate against hepatitis B and C could go a long way to prevent liver cancer. Main initiatives should focus on increasing awareness and free screening in communities to detect and treat cases at an early stage. Additionally, making vaccination against hepatitis B a routine immunisation at birth and freely available throughout adulthood, could go a long way in combating preventable infections. At a policy level, health economic models could help governments decipher the most cost-effective strategies to reduce the liver cancer burden in their context which could include providing essential medicines as part of a national health insurance package, and making these freely available at the community level to all who need them.
